# Telerehabilitation during the COVID-19 pandemic in Sweden: a survey of use and perceptions among physiotherapists treating people with neurological diseases or older adults

**DOI:** 10.1186/s12913-022-07968-6

**Published:** 2022-04-26

**Authors:** Lucian Bezuidenhout, Conran Joseph, Charlotte Thurston, Anthea Rhoda, Coralie English, David Moulaee Conradsson

**Affiliations:** 1 Department of Neurobiology, Care Sciences and Society, Division of Physiotherapy, Karolinska Institutet, Stockholm, Sweden; 2 Faculty of Community and Health Sciences; Deanery, University of the Western Cape, Bellville, South Africa; 3 Department of Health and Rehabilitation Sciences, Division of Physiotherapy, Stellenbosch University, Cape Town, South Africa; 4 School of Health Sciences and Priority Research Centre for Stroke and Brain Injury, University of Newcastle, Newcastle, NSW Australia; 5 Medical unit Occupational therapy & Physiotherapy, Theme Women’s Health and Allied Health Professional, Karolinska University Hospital, Stockholm, Sweden

**Keywords:** Physiotherapists, Telerehabilitation, Information and Communication Technology, Perceptions, COVID-19 pandemic

## Abstract

**Background:**

Telerehabilitation, i.e. rehabilitation at a distance using Information and Communication Technology (ICT), is a promising avenue for improving health among people with neurological diseases or older adults who often experience limited access to services. Still, little is known about physiotherapists’ use, perceptions and needs with regards to telerehabilitation services.

**Aims:**

To describe physiotherapists use and perceptions of, as well as needs for, telerehabilitation services for the rehabilitation of people with neurological diseases or older adults in Sweden.

**Methods:**

In this cross-sectional study, an author-created survey was sent out to members of the Swedish Association of Physiotherapists including questions about the use and perceptions of existing telerehabilitation services (e.g. telephone, internet-based applications and mobile applications) as well as needs of future telerehabilitation services. The results were presented descriptively as numbers and percentages.

**Results:**

Three hundred seven physiotherapists were included in this study with 139 (45%) treating people with neurological diseases and 168 (55%) treating older adults. Most respondents did not provide telerehabilitation before (74%) or during (51%) the COVID-19 pandemic. Telephone, which was predominantly used for administrative tasks, was the most frequent utilised ICT used by 68% of the physiotherapist using ICTs several days/week. Few respondents used internet-based applications (12%), mobile applications (3%) or SMS services (8%) and videoconferencing (3%). A majority of the respondents were interested in ICT (78%), felt comfortable using ICT (57%) and were interested in learning how ICT can be used in rehabilitation (92%). Still, few respondents perceived that people with neurological diseases or older adults can use existing ICTs for rehabilitation purposes (18%) and that existing reimbursement system within health care facilitates remote rehabilitation (16%). Important functionality of future ICT perceived by physiotherapists covered patient communication (e.g. chat, SMS and video), assessments (e.g. digital surveys and assessment of physical activity) and treatment (e.g. exercise prescription).

**Conclusion:**

While physiotherapists had an overall positive perception to use and willingness to learn about telerehabilitation, few used telerehabilitation services before nor during the COVID-19 pandemic and they also perceived multilevel barriers for implementation, ranging from patients ability to use ICT to existing reimbursement systems within health care. Our findings emphasize the need to strengthen the expertise regarding remote services among physiotherapists.

**Supplementary Information:**

The online version contains supplementary material available at 10.1186/s12913-022-07968-6.

## Introduction

The COVID-19 pandemic has had devasting consequences globally with most healthcare systems being challenged [1–3]. It has also become clearer that the effect of the pandemic goes beyond the diseases it produces, as healthcare systems at the same time have to cope with the rehabilitation needs among those with pre-existing comorbidities. These challenges are more prominent among those living with disability, multimorbidity and frailty, e.g. due to neurological diseases [4, 5] or old age [3]. These groups are more dependent on regular rehabilitation services to sustain function, physical activity and quality of life [1, 6, 7] and often experience limited access to rehabilitation due to problems with transportation to the clinic [8, 9], particularly those with lower incomes [9]. The COVID-19 pandemic has also contributed to new barriers for access to rehabilitation since many components of rehabilitation require patient contact, e.g. supervised balance exercises or strength training, and such services are therefore prone to cancelation due to the risk of spreading the infection [10]. For those patients with ongoing rehabilitation needs, the inevitable decisions of physical distancing for protection of both healthcare workers and the general public could contribute to increases in disability and morbidity from a lack of necessary rehabilitation services [10].

To counter these barriers, proactive strategies need to be developed to provide accessible rehabilitation services remotely. Telerehabilitation, i.e. the delivery of rehabilitation services at a distance using Information and Communication Technology (ICT), is a promising avenue to improve access to rehabilitation [11, 12]. Information and Communication Technology includes a range of telecommunication tools and technology (e.g. telephone, internet-based applications and mobile applications) and its associated software, which could be used to support telerehabilitation by enabling real-time consultations, assessments and intervention remotely e.g. through text message and video conferencing [11, 13]. The goal of telerehabilitation is to deliver services remotely to clients in their homes or other living environments and thereby improve access to rehabilitation. The therapist-patient interaction may take place in real-time (e.g. video meetings) or asynchronously (i.e. communication or data collected at one time point and interpreted or responded to later). Within the field of physiotherapy, telerehabilitation could be used as a complimentary service to regular rehabilitation and previous studies support the feasibility of using ICT to support remote contact and treatment of older adults [14] and people with neurological diseases [15, 16]. Until now, implementation of telerehabilitation into existing rehabilitation models has been slow – especially services to people with a disability (e.g. neurological disease) or old age [16, 17]. Reported barriers to learn and use new ICTs among people aged ≥ 65 years during the pandemic are increased age, low level of education and self-reported fair or poor general health [18]. Furthermore, people with neurological disease or old age might have cognitive, visual and fine motor impairments affecting their ability to manage ICT, e.g. difficulties with reading the font on a screen, orienting the user-interfaces and manual handling of the device [17, 19–24].

The Swedish Association of Local Authorities and Regions have created a vision (Vision e-Health 2025) to become a global leader in the utilization of e-Health solutions by 2025 [25]. However, despite a general increase in remote healthcare consumption in Sweden during the first wave of the pandemic [26], it is not known if this also is reflected in telerehabilitation. Experiences from the perspective of the patients rather indicates that rehabilitation services were reduced during the first and second wave of the COVID-19 pandemic. For instance, 80% of the members of NEURO Sweden (a patient organization for people with neurological diseases in Sweden) reported cancelled rehabilitation services during 2020 and < 10% had been offered services remotely [27]. With the uncertainty regarding the future of the COVID-19 pandemic, as well as the potential of telerehabilitation to improve access to specialist care beyond the need during pandemics, it is important that the rehabilitation services for older adults and people with neurological diseases adapt to more remote models.

Still, there is a knowledge gap in the use and perception of telerehabilitation among physiotherapists treating patients with neurological diseases or old age. Understanding of the utility and perception of ICT for promoting telerehabilitation can help guide effective planning for the uptake of telerehabilitation into practice. Therefore, the overarching aim of this study was to gain insight into the use and perceptions of telerehabilitation and ICTs among physiotherapists treating people with neurological diseases and older adults in Sweden. The specific aims were to describe physiotherapists’: 1) use of telerehabilitation both before and during the COVID-19 pandemic, 2) use of ICT during the pandemic, 3) perceptions of different types of ICTs and telerehabilitation services, and 4) perceptions of needs of telerehabilitation services.

## Methods

### Participants

An online author-created survey was sent by email to physiotherapists registered as members of the Neurology, Health of the Elderly, and Primary Care groups of the Swedish Association of Physiotherapists in September 2020 (i.e. initial phase of the second wave of the COVID-19 pandemic in Sweden). Members of these groups are most likely to treat older adults and people with neurological diseases in a range of Swedish care settings (i.e. in hospitals, primary care facilities and the community). Of the total number of registered physiotherapists in Sweden (approximately 16 000), about 3000 physiotherapists are members of the three targeted groups. Physiotherapists who worked clinically and treated either patients with neurological diseases or geriatric patients were included in this study. The rational for using a digital survey to investigate physiotherapists’ perceptions of telerehabilitation and ICTs was the opportunity to reach a larger group of the targeted population in a resource-efficient and fast way. This study was approved by the Regional Board of Ethics in Stockholm (2020–01,850) and all participants provided informed consent through the online survey before being given access to the questions included in the survey.

### Construction of the web-survey

The development of the survey was adapted by the research group from a previous survey on provision of physiotherapy for people with Parkinson’s Disease in Sweden [28]. While the questions regarding demographics and response categories used in this study were similar to those used for the survey for provision of physiotherapy for Parkinson’s Disease, additional questions were included to investigate physiotherapists’ perceptions of telerehabilitation and ICTs for this study. These questions regarding physiotherapists perceptions of telerehabilitation and ICTs were motivated by the NASSS (non-adoption, abandonment, scale-up, spread, sustainability) framework [29]. The NASSS framework describes ICTs as part of a complex dynamic system and focuses on the use, value and perception of technology among end-users. The survey was initially tested for content validity, including feasibility, readability and presentation among 15 physiotherapists with clinical and research experience in geriatric and neurological rehabilitation, with modifications made based on feedback. As an example, based on the feedback from the physiotherapists reviewing the initial survey, we decided to refer to telerehabilitation as the delivery of rehabilitation services at a distance using “digital tools” to support telerehabilitation services in the survey. The term “digital tools” was used in the survey instead of ICT since this term was more familiar to physiotherapists in Sweden.

### Procedure: web-survey

An email which included a hyperlink to a web-survey was sent to each participating physiotherapist. The web-survey took approximately ten minutes to complete and respondents were required to choose from multiple choice answers. The web-survey was available for approximately five weeks during September of 2020 and three emails were sent as a reminder during this period.

### Content of the survey and data management

The initial questions of the web-survey collected information regarding demographics (sex and age), main patient group treated (i.e. geriatric patients, patients with neurological diseases or other), work setting (i.e. hospital, primary care unit, community center or private), work status (full time or part time work), years of work experience and highest level of education. Response categories for age were: < 30, ≥ 30–39, ≥ 40–49, ≥ 50–59 and ≥ 60 years and work experience were: ≤ 5, 6–10 and > 10 years. Categories for highest education were: bachelor degree, master of science, clinical specialist and doctor of philosophy. In the Swedish context, a clinical specialist is a physiotherapist with a master of science degree with at least five years of work experience including three years of supervised practice within the area of specialization.

The remainder of the survey was divided into 4 domains (detailed in supplementary material 1) focusing on the use and perceptions of telerehabilitation and ICTs. The first domain focused on the proportion of patients treated remotely before and during the COVID-19 pandemic. The second domain addressed the use of ICT in clinical work during the pandemic. The physiotherapists who reported that they used a specific ICT frequently (i.e. several days/week), were also asked about the purpose of using the ICT during the pandemic (i.e. patient appointment booking, history taking, assessments, prescription of exercise program, advice and information, and follow-up of treatment). The third domain addressed the physiotherapists perceptions of telerehabilitation and ICTs using close ended questions. The first section of this domain addressed how much time each day the physiotherapists would consider devoting to telerehabilitation. The second part of this domain addressed the physiotherapists perceptions of using computer and mobile phone-based ICTs from: 1) the physiotherapist perspective (e.g. interest, technology use and value), 2) the patients they treat (e.g. usability, appreciation and accessibility) and 3) the workplace (e.g. accessibility, collegial support and financial reimbursement). The fourth domain addressed the required functions of ICT which are perceived to be important by physiotherapist to support telerehabilitation. The Information and Communication Technology functions were related to patient communication, assessment and treatment.

Statistical analyses were carried out using IBM SPSS, version 23.0 (SPSS Inc., Chicago, Illinois, USA). The data obtained from the web-survey were categorized as either binominal or ordinal and presented descriptively as numbers and percentages for each category (see supplementary material 1). The results were presented separately for physiotherapists who primarily treated either people with neurological diseases or older adults since these patient groups might have different abilities to use ICTs and engage in telerehabilitation, and ICT might therefore be used differently by physiotherapists.

## Results

### Response and sample characteristics

Of the approximately 3000 targeted physiotherapists, 385 physiotherapists (12.8% response rate) completed the survey. Of the 385 physiotherapists which responded to the survey, 307 were included in this study with 139 (45%) treating people with neurological diseases and 168 (55%) treating older adults. We excluded 68 physiotherapists who treated other patient groups and 10 physiotherapists who were not working clinically. Table 1 presents sex, age, work setting, work status, work experience and educational level of the respondents. The majority of the respondents were female (neurology: *n* = 128, 93%, geriatrics: *n* = 149, 89%), worked full time (neurology: *n *= 101, 74%, geriatrics: *n* = 120, 72%), reported > 10 years of clinical experience (neurology: *n* = 106, 76%, geriatrics: *n* = 129, 77%) and had a bachelor degree in physiotherapy as their highest education (neurology: *n* = 93, 67%, geriatrics: *n* = 146, 87%). The most common work setting was hospitals (*n* = 87, 63%) for physiotherapists working with people with neurological diseases, and community services (*n* = 117, 69%) for the majority of physiotherapists working with older adults.

### Use of telerehabilitation before and during the COVID-19 pandemic

**Fig. 1:**
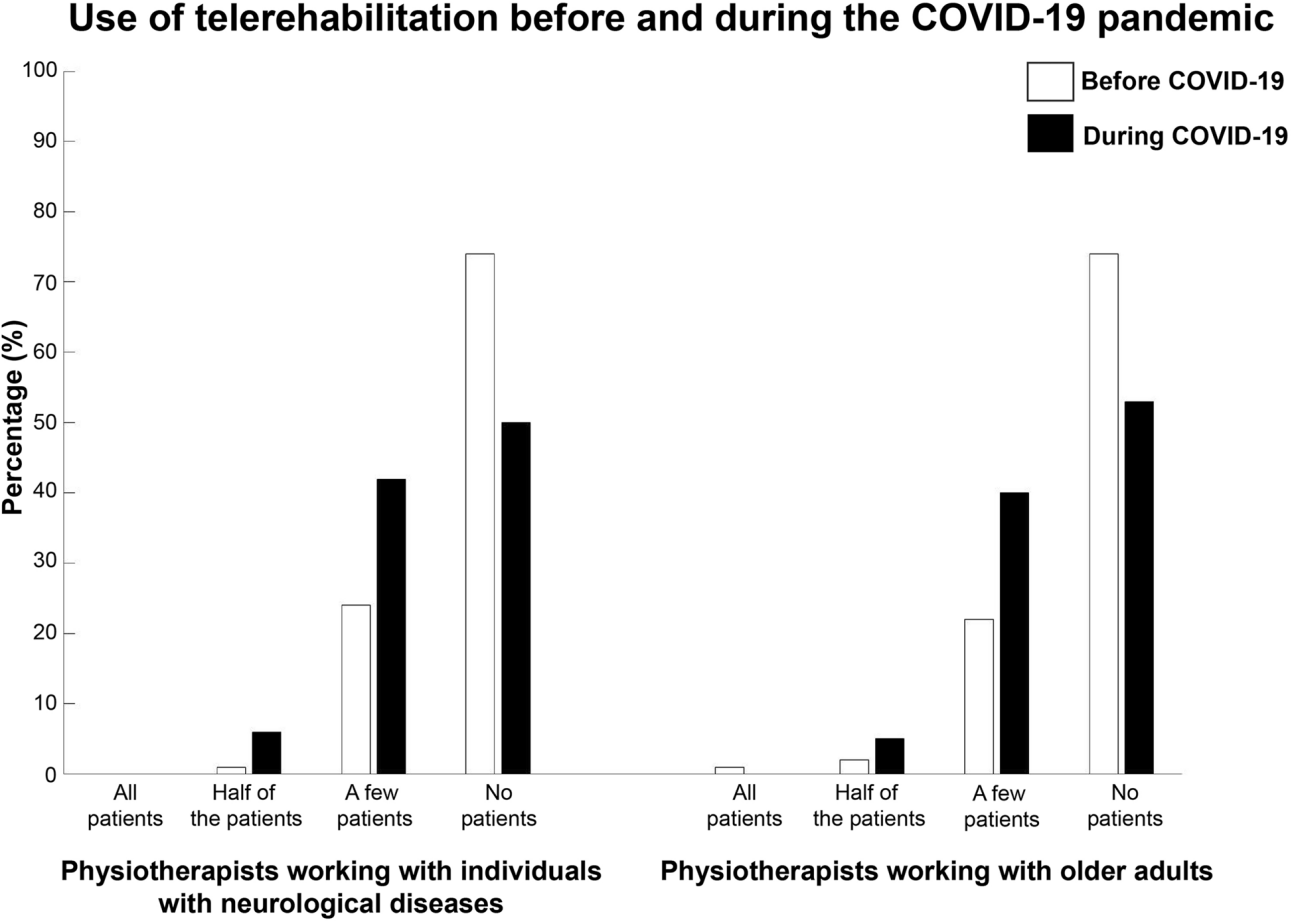
Use of telerehabilitation among physiotherapists working with individuals with neurological diseases and older adults before and during the COVID-19 pandemic

As shown in Fig. 1, most respondents did not provide telerehabilitation services before (neurology: *n* = 103, 74%, geriatrics: *n* = 125, 74%) or during (neurology: *n = *69, 50%, geriatrics: *n* = 89, 53%) the COVID-19 pandemic. More respondents indicated they had provided telerehabilitation to “a few patients” during the pandemic (neurology: *n* = 59, 42%, geriatrics: *n* = 67, 40%) compared with before the pandemic (neurology: *n* = 33, 24%, geriatrics: *n* = 37, 22%).

### Use of Information and Communication Technologies during the COVID-19 pandemic

Use of ICT are presented in Table 2 and the purpose of using the ICT is detailed in supplementary material 2. The most commonly used ICT in the provision of telerehabilitation services was telephone (neurology: *n* = 80, 57%, geriatrics: *n* = 135, 80%, Table 2). Telephone services were primarily used for administrative tasks, e.g. patient appointment bookings (neurology: *n* = 70, 88%, geriatrics: *n* = 125, 93%), followed by follow-ups of treatment (neurology: *n* = 72, 90%, geriatrics: *n* = 122, 90%) and advice and information (neurology: *n* = 50, 63%, geriatrics: *n* = 77, 57%). Internet-based applications (neurology: *n* = 12, 8%, geriatrics: *n* = 28, 17%) and SMS services (neurology: *n* = 17, 12%, geriatrics: *n* = 28, 5%) were used to a lesser extent by the respondents. SMS-services were exclusively used for booking patient appointments and internet-based applications were predominantly used for the prescription of exercise programs (neurology: *n* = 10, 83%, geriatrics: *n* = 25, 89%). Very few respondents used either video conferencing (neurology: *n* = 6, 4%, geriatrics: *n* = 4, 2%) or mobile applications (neurology: *n* = 1, 1%, geriatrics: *n* = 9, 5%).

### Physiotherapists’ perceptions of telerehabilitation and Information and Communication Technologies

**Fig. 2:**
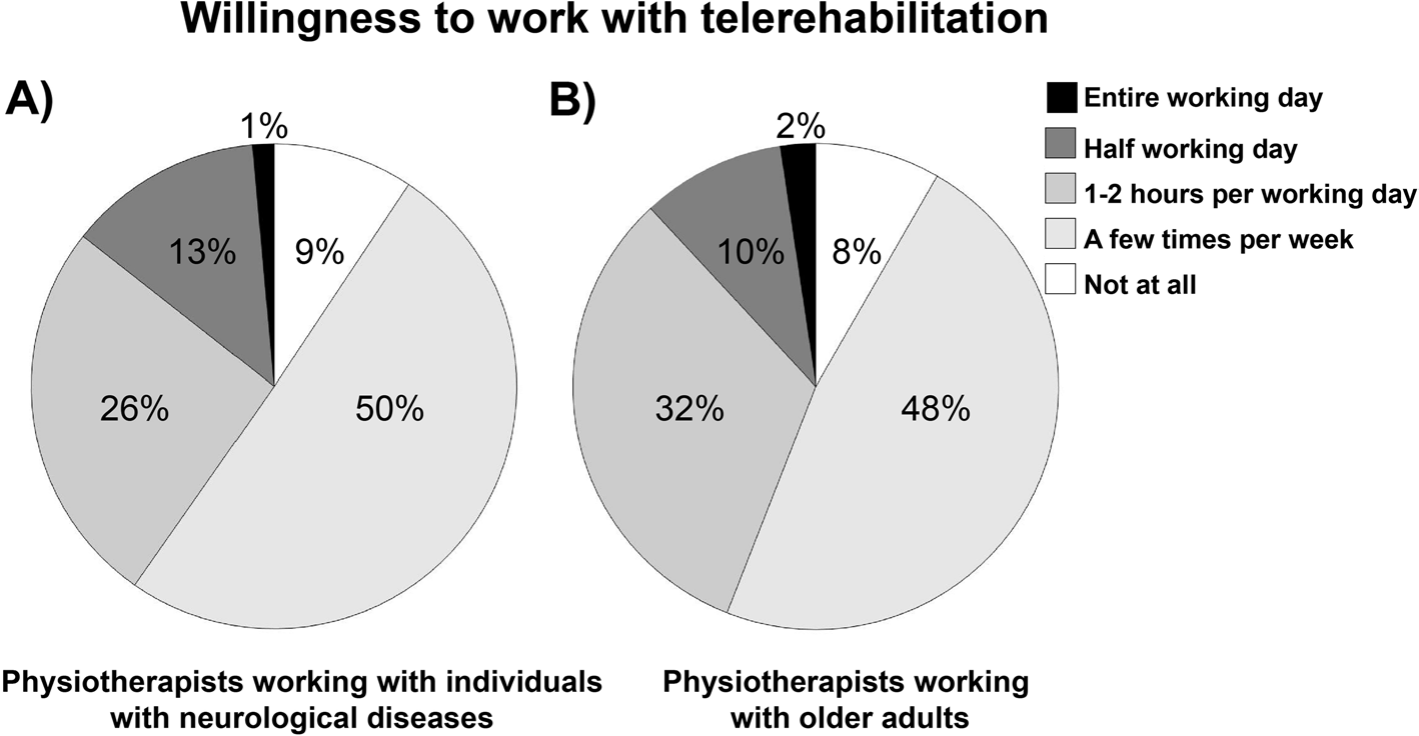
Willingness to work with telerehabilitation among **A** physiotherapists working with individuals with neurological diseases and **B** physiotherapists working with older adults

As shown in Fig. 2, about half of the respondents (neurology: *n* = 70, 50%, geriatrics: *n* = 80, 48%) were willing to work with telerehabilitation a few times per week whereas about one in ten of the respondents (neurology: *n* = 13, 9%, geriatrics: *n* = 14, 8%) were not willing to work with telerehabilitation at all.

**Fig. 3:**
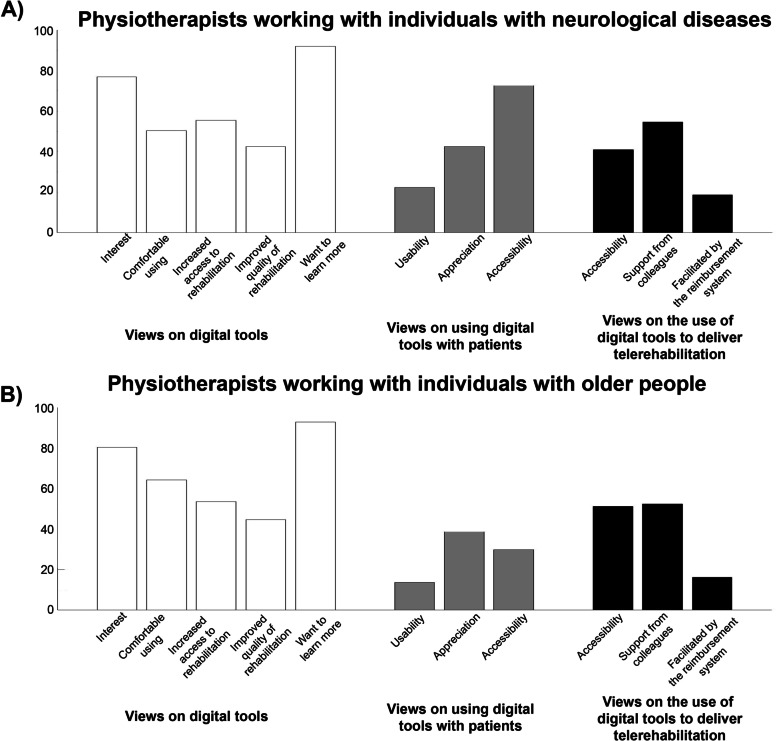
Perceptions of telerehabilitation and digital tools among **A** physiotherapists working with patients with individuals with neurological diseases **B** and physiotherapists working with older adults. The results are presented from the physiotherapist views of using digital tools, the perspectives of using digital tools with the patients they treat and their views of digital tools to deliver telerehabilitation

As illustrated in Fig. 3, a majority of the respondents were interested in (neurology: *n* = 107, 77%, geriatrics: *n* = 135, 80%) and felt comfortable (neurology: *n* = 70, 50%, geriatrics: *n* = 108, 64%) using ICTs and were interested in learning how ICT can be used in rehabilitation (neurology: *n* = 128, 92%, geriatrics: *n* = 156, 93%). Most respondents perceived that ICTs would improve accessibility to rehabilitation (neurology: *n* = 77, 55%, geriatrics: *n* = 90, 54%) and about four of ten perceived that ICT would improve the quality of rehabilitation (neurology: *n* = 59, 42%, geriatrics: *n* = 75, 45%). Few respondents (neurology: *n* = 31, 22%, geriatrics: *n* = 23, 14%) perceived that people with neurological diseases or older adults can use existing ICTs (e.g. computer, tablet, or mobile application) for telerehabilitation purposes. The majority of respondents working in neurology (*n* = 101, 73%) perceived that people with neurological diseases have access to a computer, tablet or mobile phone, compared with 30% (*n* = 50) of respondents working with older adults. A minority of the respondents (neurology: *n* = 24, 17%, geriatrics: *n* = 23, 13%) felt that the existing reimbursement system that applies to their workplace facilitates telerehabilitation.

### Physiotherapists’ needs of telerehabilitation services

**Fig. 4:**
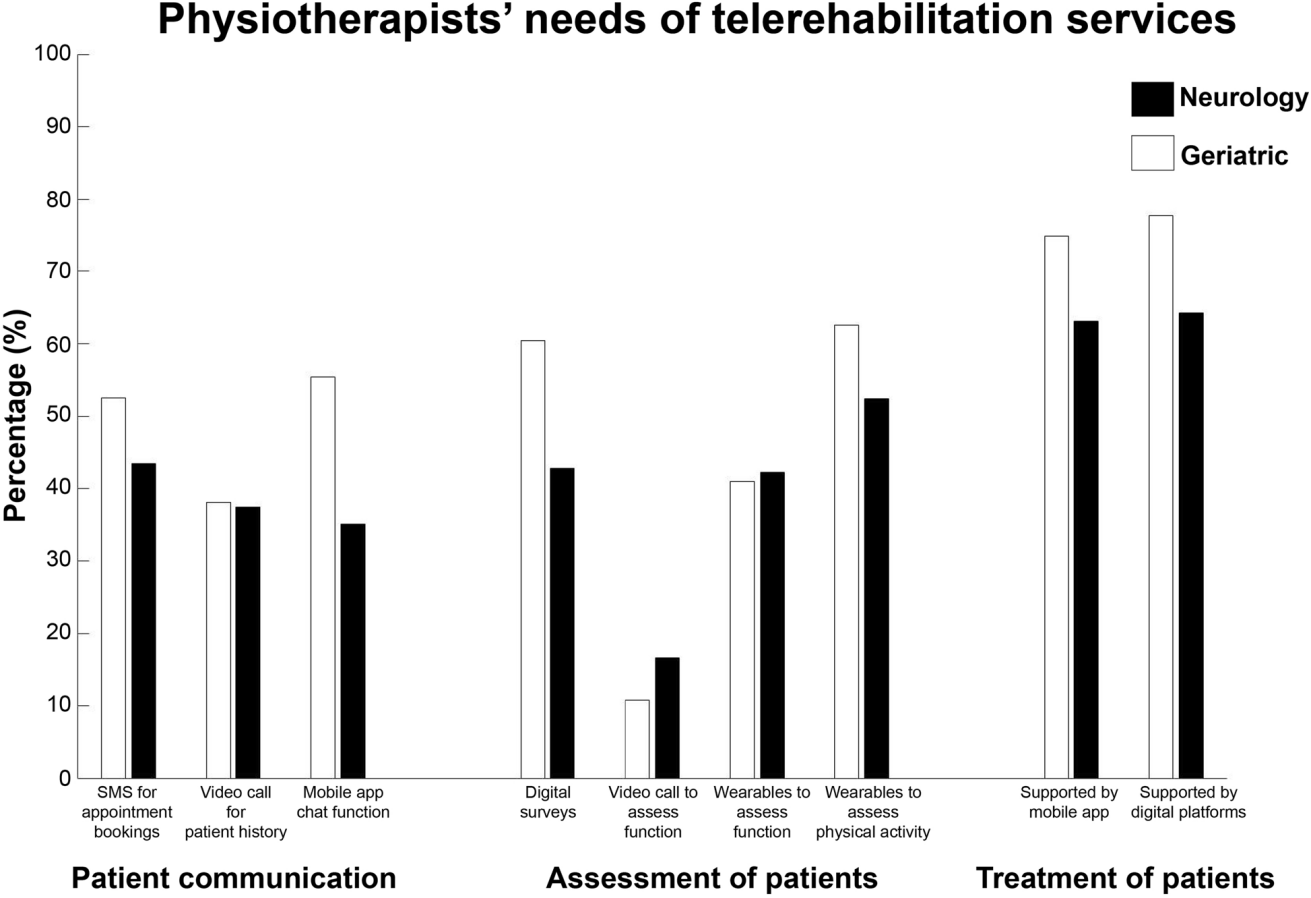
Physiotherapists’ perceptions of needs of telerehabilitation services with respect to patient communication, assessment and treatment

Respondents treating people with neurological diseases perceived patient appointments via SMS services (*n* = 73, 53%), patient-therapist communication using chat functions (*n* = 77, 55%), digital surveys (*n *= 84, 60%), objective measurement of physical activity using wearables (*n* = 87, 63%), prescription of home exercises and regimes using mobile applications (*n* = 104, 75%) and internet-based systems (*n* = 108, 78%) to be important functions for ICTs (Fig. 4). Physiotherapists treating older adults reported objective measurement of physical activity using wearables (*n* = 89, 52%), prescription of home exercises and regimes using mobile applications (*n* = 107, 63%) or internet-based systems (*n* = 109, 64%) to be important.

## Discussion

The present study sheds light on the use and perceptions of telerehabilitation services among physiotherapists treating people with neurological diseases and older adults in Sweden. The results showed that most of the physiotherapists did not provide telerehabilitation services either before or during the COVID-19 pandemic. In line with findings from previous studies [22, 30–32], the responding physiotherapists in our study perceived that limited abilities using existing ICTs among people living with neurological diseases or older adults and existing reimbursement system within health care restricted the use of telerehabilitation. Despite this, the majority of physiotherapists perceived ICT to be important and were interested to learn more about how to use ICTs to support telerehabilitation of people with neurological diseases and older adults. Our findings emphasize the need to develop and implement ICTs reflecting the needs of physiotherapists and people with neurological diseases and old age, and to strengthen the expertise among physiotherapists with regards to remote assessment and treatment of patients.

While the current evidence regarding the effectiveness of telerehabilitation for patients with neurological diseases and old age is inconclusive [11, 14, 16], the field is growing with numerous ongoing clinical trials. In line with the present results as well as socio-technical theory domain of the NASSS framework [29], the utilization of ICT to support telerehabilitation is often influenced by several factors (e.g. therapist, patient and organization). Hence, there is a risk that the benefits of telerehabilitation (e.g. time and cost efficiency, accessibility and convenience) are outweighed by technical, patient-related and organizational barriers. For instance, the attitudes among physiotherapists towards ICT may influence the implementation of telerehabilitation. In line with previous findings [19, 33], most of the physiotherapists in our study had a positive attitude towards ICTs, perceived telerehabilitation to be valuable for practice and were interested to learn more about how to use ICTs to support telerehabilitation. We believe this reflects the changing landscape in terms of ICT use broadly in society, and more particularly the interest in professional development among physiotherapists and the awareness to adapt clinical practice to accessible rehabilitation services remotely during the COVID-19 pandemic. However, despite the great need of telerehabilitation during the pandemic, only 40% of the physiotherapists treating people with neurological diseases and 44% treating older adults were willing to work with telerehabilitation on a regular basis (i.e. at least 1–2 h per day). This combined with the limited experience of telerehabilitation among the physiotherapists in our study and the perception that patients are not capable of using ICTs likely reflects important barriers to implementation of telerehabilitation in Sweden.

Brouns et al. (2019) reported that willingness to work with telerehabilitation among healthcare professionals post-stroke was positively influenced by perceived patient benefits (e.g. reduced travel time, increased motivation, better outcomes) and negatively influenced by insufficient knowledge about ICT [21]. Previous studies have also reported concerns among health-care professionals working in neurology or geriatrics that the increased use of ICT would reduce the face-to-face contact with patients and thereby reducing the establishment of a relationship with the patient [22, 33]. The physiotherapists in our study reported concern that their patients would not have the ability to use technology for remote rehabilitation services. On the other hand, most respondents perceived that ICTs would improve accessibility to rehabilitation for these populations. This is an interesting conflict of perception which might reflect the perception of physiotherapists working with people with neurological diseases and old age on their patient’s ability to manage ICTs, due to technological barriers (e.g. handling devices) and/or cognitive impairments. This concern has been reported previously among health care professionals [19–22]. For instance, cognitive impairments and impaired fine motor skills are examples of impairments which might restrict the use of ICTs in people with neurological disease [19–23]. However, this concern may be erroneous since recent studies have demonstrated patient engagement with telerehabilitation during the COVID-19 pandemic [34, 35]. Qualitative studies have also reported that people with neurological diseases and older adults perceive telerehabilitation as convenient and motivating, easy to cope with the technology and that positive therapeutic relationships are possible [36, 37].

It is important to acknowledge that healthcare professionals perception of acceptability of ICT does not reflect the patient view and therefore it is necessary to explore the patients view directly. It is also important to highlight that the use and uptake of ICT and telerehabilitation models is not solely dependent on the accessibility and usability of ICTs it is also dependent on how it is introduced, delivered and supported. The physiotherapists in the present study emphasized the importance of factors related to the organizational context (i.e. existing care agreement and system for financial compensation) in implementing ICT and remote services – which is in line with previous studies [22, 38].

In our study, telephone was the most used ICT by physiotherapists for administrative tasks (i.e. patient appointment booking) and history taking, advice and information and follow-up of treatment, which is in line with previous research [16, 39]. While the advantages of using a telephone is its low cost and availability at all workplaces, relying only on verbal information for therapist-patient communication could be a limitation. In contrast, internet-based applications and mobile applications offering several functions for communication (e.g. video and chat), exercise (e.g. supervised online or prescribed) and follow-up assessments (e.g. digital surveys) has the potential to remotely support all aspects of standard in-person rehabilitation services. While the physiotherapists in our study perceived advantages and needs for ICT with a diverse technical functionality for delivery of remote care, few respondents had any experience in their use.

Delivering rehabilitation in the same way as before the pandemic will neither be practical nor meet the increased need of rehabilitation in the future. Telerehabilitation has great potential to be an important complement to conventional rehabilitation (i.e. face-to-face visits) to manage the challenges of the future with an aging population, limited resources and increased demands for accessibility, let alone future pandemics [25]. To accomplish this, it is important that ICTs are appropriate and accessible to people living with neurological diseases and old age, and not disadvantage some groups, e.g. those with greater levels of disability, cognitive impairment, and/or limited social support. Previous studies regarding the uptake of telerehabilitation in the United States during COVID-19 pandemic highlight this issue. These results showed that those who received telerehabilitation services were more likely to be young and live in larger metropolitan areas [34, 40]. For clinical practice, this survey indicates a need among physiotherapists in Sweden to professionally develop regarding ICTs and telerehabilitation services. For uptake of ICT among physiotherapists, it appears crucial that ICTs are user-friendly, but also fit seamlessly into existing processes of care. Since the uptake of telerehabilitation services starts with the therapists introducing technology for rehabilitation purposes to the patients [41], the factors affecting the utility of ICTs addressed by physiotherapists in our study should be an important starting point to increase the uptake of telerehabilitation. Barriers related to patient capability of using ICT may be reduced by tailoring instructions for using ICT to the clients’ capacity and preferences and through comprehensive training and accessible support [42, 43]. We encourage future investigations of barriers for telerehabilitation and ICTs perceived by physiotherapist and patients, as well as studies of the link between technical expertise, attitudes and clinical decision making among physiotherapists treating patients with neurological diseases and older adults. It is also important to emphasize the need to better understand organizational readiness for telerehabilitation and how to support physiotherapists on implementing remote working models for rehabilitation.

### Study strengths and limitations

This study has both strengths and limitations. Our sample size of just over 300 physiotherapists, while small, represents about 10% of the targeted 3000 members of the Neurology, Health of the Elderly and Primary care sections of the Swedish Association of Physiotherapists. However, the distribution of gender, age, work experience and highest level of education were similar in the present study sample and a previous survey including a larger sample (*n* = 705) of physiotherapists working in neurology and geriatrics [28]. We acknowledge the methodological limitations of surveys and that responses may not necessarily reflect actual practices. Future studies should also include people living with neurological diseases or old age to capture their needs and perceptions of telerehabilitation services.

## Conclusion

Most physiotherapists treating people with neurological diseases or older adults in Sweden did not use telerehabilitation services before nor during the COVID-19 pandemic. To support telerehabilitation and the vision of Swedish Association of Local Authorities and Regions to become a global leader in the utilization of e-Health solutions by 2025, our findings emphasize the need to strengthen the expertise regarding remote assessments and treatments among physiotherapists working with people with neurological diseases and older adults. To establish and offer evidence-based models for telerehabilitation, education courses and programs needs to be provided to physiotherapists in order to improve the accessibility of rehabilitation services to people with neurological disease or older adults. We also encourage future studies of barriers for telerehabilitation and ICTs, as well as studies between the link of technical expertise, attitudes and clinical decision making among physiotherapists treating patients with neurological diseases and older adults.

**Table 1:** Demographics of the survey participants

**Variables, *n* (%)**	**Neurology** (*n *= 139)	**Geriatrics** (*n *= 168)
Female	128 (93)	149 (89)
Age		
< 30 years	6 (4)	9 (5)
≥ 30–39 years	33 (24)	41 (24)
≥ 40–49 years	33 (24)	46 (27)
≥ 50–59 years	49 (35)	57 (34)
≥ 60 years	18 (13)	15 (9)
Work setting		
Hospital	87 (63)	9 (5)
Primary care	37 (26)	40 (24)
Community	14 (10)	117 (70)
Private	1 (1)	2 (1)
Work status		
Full time work	101 (74)	120 (72)
Part time work	36 (26)	46 (28)
Working experience		
1–5 years	10 (7)	19 (11)
6–10 years	23 (17)	20 (12)
> 10 years	106 (76)	129 (77)
Education		
Bachelor degree	93 (67)	146 (87)
Clinical specialist	25 (18)	7 (4)
Master of science	15 (11)	12 (7)
Doctor of philosophy	6 (4)	3 (2)

**Table 2:** Use of Information and Communication Technologies among physiotherapists working in neurology and geriatrics during the COVID-19 pandemic

**Variables, n (%)**	**Neurology** (*n* = 139)	**Geriatrics** (*n* = 168)
Telephone	80 (57)	135 (80)
SMS services	17 (12)	8 (5)
Video conferencing	6 (4)	4 (2)
Internet-based application	12 (8)	28 (17)
Mobile applications	1 (1)	9 (5)

## Supplementary Information

**Additional file 1: Supplementary material 1. **Domains, item descriptions and responses included inthe four sections of the survey. **Supplementary material 2. **Number (percentage) ofphysiotherapists working with in neurology and geriatric who reported that they used Information andCommunication Technologies frequently (i.e. several days/week) duringthe COVID-19 pandemic and the purposeof using the ICT. Multiple answers were allowed for the purpose of usingICTs. 

## Data Availability

Since data can indirectly be traced back to the study participants, according to the Swedish and EU personal data sharing legislation, access can only be granted upon request. The request should be addressed to Cecilia Martinsson Björkdahl (rdo@ki.se) who is the institutional contact handling data access requests at Karolinska Institutet. Any sharing of data will be regulated via a data transfer and use agreement with the recipient and require ethical approval from the Regional Board of Ethics in Stockholm.

## Abbreviation

ICT: Information and Communication Technology
