# Supplementary material for: Telerehabilitation during the COVID-19 pandemic in Sweden: a survey of use and perceptions among physiotherapists treating people with neurological diseases or older adults
Source: BMC Health Serv Res. 2022 Apr 26;22:555. doi: 10.1186/s12913-022-07968-6 (PMC9038993; doi:10.1186/s12913-022-07968-6)
Supplement: Supplementary file 1 — Additional file 1: Supplementary material 1. Domains, item descriptions and responses included inthe four sections of the survey. Supplementary material 2. Number (percentage) ofphysiotherapists working with in neurology and geriatric who reported that they used Information andCommunication Technologies frequently (i.e. several days/week) duringthe COVID-19 pandemic and the purposeof using the ICT. Multiple answers were allowed for the purpose of usingICTs. [file 12913_2022_7968_MOESM1_ESM.docx]

| **Supplementary material 1.** Domains, item descriptions and responses included in the four sections of the survey. | | |
| --- | --- | --- |
| **Domain** | **Item description** | **Response** |
| ***1: Provision of telerehabilitation before and during the COVID-19 pandemic*** | | |
| Tele-rehabilitation | **A:** *Which statement about telerehabilitation best reflects your work with patients before the covid-19 pandemic?*  **B:** *Which statement about telerehabilitation best reflects your work with patients during the covid-19 pandemic?* | **A-B:**  1) All rehabilitation was/is performed remotely  2) About half of the patients were/are treated remotely  3) A few patients were/are treated remotely  4) No patients were/are treated remotely |
| ***2: Use of digital tools*** | | |
| Digital tools | **A:** *How often do you use telephone/SMS services /video conferencing/* *internet-based application /mobile applications in patient work?*  **B:** *For what purpose do you use telephone/SMS services /video conferencing/* *internet-based application /mobile applications in patient work?* | **A:**  1) Every day  2) Several days per week  3) Several days per month  4) Never  Reponses 1-2 indicated regular utilization of digital tools.  **B:**  1) Patient appointment booking  2) Patient history taking  3) Assessments (clinical tests/ questionnaires)  4) Prescription of exercise program  5) Advice and information  6) Follow-up of treatment  Multiple answers allowed. |
| ***3: Perceptions of telerehabilitation and digital tools*** | | |
| Willingness | *How much of your work day would you consider devoting to telerehabilitation using digital tools?* | 1) Entire working day  2) Half working day  3) 1-2 hours per working day  4) A few times per week  5) Not at all |
| Perceptions | **Physiotherapist perspective**  **A:** *I am interested using digital tools (e.g. computer, tablet, or mobile phone) for telerehabilitation*  **B:** *I am comfortable using digital tools (e.g. computer, tablet, or mobile application) for telerehabilitation*  **C:** *I believe that digital tools will increase the accessibility of rehabilitation for the patients I treat*  **D:** *I believe that digital tools will improve the quality of rehabilitation for the patients I treat*  **E:** *I want to learn how new digital tools can be used in rehabilitation*  **Patient perspective**  **F:** *Most patients I treat can use digital tools (e.g. computer, tablet, or mobile application) in their rehabilitation*  **G:** *I think patients will appreciate telerehabilitation*  **H:** *Most patients I treat have access to a computer, tablet, or mobile phone*  **Work place**  **I:** *My workplace has access to digital tools for telerehabilitation*  **J:** *I perceive support from my colleagues and my boss regarding new digital tools for telerehabilitation*  **K:** *The reimbursement system that apply to my workplace facilitate telerehabilitation with the support of digital tools* | **A-L:**  1) Totally agree  2) Agree somewhat  3) Disagree somewhat  4) Not correct at all  Reponses 1-2 indicated a positive perception regarding digital tools/telerehabilitation. |
| **4: Needs of telerehabilitaton services** | | |
|  | *To what extent do you think the following functions of a digital tool are important for patient work*?  **Patient communication**  **A:** *Patient appointment bookings via text message would facilitate patient work*  **B:** *Patient history taking using video calls would facilitate patient work*  **C:** *Chat function via mobile application would facilitate patient work*  **Assessment of patients**  **D:** *Digital surveys or questionnaires would facilitate patient work*  **E:** *Functional assessment (e.g. clinical tests) using video calls would facilitate patient work*  **F:** *Objective measurements of the patient's functional ability (e.g. movement quality and gait patterns) via wearables integrated in a mobile application would facilitate patient work*  **G:** *Objective measurements of the patient's physical activity levels via wearables integrated in a mobile application would facilitate patient work*  **Treatment of patiens**  **H:** *Treatment support via mobile application (e.g. prescription of home exercises and regimes) would facilitate patient work*  **I:** *Treatment support via digital platforms (e.g. prescription of home exercises and regimes) would facilitate patient work* | **A-I**:  1) Totally agree  2) Agree somewhat  3) Disagree somewhat  4) Not correct at all  Reponses 1-2 indicated that the function for digital tools was needed for telerehabilitation. |

| **Supplementary material 2.** Number (percentage) of physiotherapists working with in neurology and geriatric who reported that they used Information and Communication Technologies frequently (i.e. several days/week) during the COVID-19 pandemic and the purpose of using the ICT. Multiple answers were allowed for the purpose of using ICTs. | | |
| --- | --- | --- |
|  | **Study sample** | |
|  | **Neurology** (n=139) | **Geriatric** (n=168) |
| **Use of telephone** | 80 (57) | 135 (80) |
| Patient appointment booking | 70 (88) | 125 (93) |
| Patient history taking | 24 (30) | 66 (49) |
| Assessments (clinical tests/questionnaires) | 5 (6) | 5 (4) |
| Prescription of exercise program | 9 (11) | 8 (6) |
| Advice and information | 50 (63) | 77 (57) |
| Follow-up of treatment | 72 (90) | 122 (90) |
| **Use of SMS services** | 17 (12) | 8 (5) |
| Patient appointment booking | 17 (100) | 8 (100) |
| Patient history taking | 0 (0) | 1 (13) |
| Assessments (clinical tests/questionnaires) | 0 (0) | 0 (0) |
| Prescription of exercise program | 0 (0) | 0 (0) |
| Advice and information | 0 (0) | 1 (13) |
| Follow-up of treatment | 1 (6) | 2 (25) |
| **Use of video conferencing** | 6 (4) | 4 (2) |
| Patient appointment booking | 0 (0) | 0 (0) |
| Patient history taking | 4 (67) | 2 (50) |
| Assessments (clinical tests/questionnaires) | 2 (33) | 0 (0) |
| Prescription of exercise program | 3 (50) | 0 (0) |
| Advice and information | 4 (67) | 0 (0) |
| Follow-up of treatment | 4 (67) | 2 (50) |
| **Use of internet-based application** | 12 (8) | 28 (17) |
| Patient appointment booking | 4 (33) | 10 (36) |
| Patient history taking | 3 (25) | 1 (4) |
| Assessments (clinical tests/questionnaires) | 6 (50) | 1 (4) |
| Prescription of exercise program | 10 (83) | 25 (89) |
| Advice and information | 6 (6) | 6 (21) |
| Follow-up of treatment | 4 (33) | 6 (21) |
| **Use of mobile applications** | 1 (1) | 9 (5) |
| Patient appointment booking | 0 (0) | 1 (11) |
| Patient history taking | 0 (0) | 2 (22) |
| Assessments (clinical tests/questionnaires) | 0 (0) | 1 (11) |
| Prescription of exercise program | 0 (0) | 4 (44) |
| Advice and information | 1 (100) | 3 (33) |
| Follow-up of treatment | 0 (0) | 1 (11) |
